# Association of Liver Transaminase Levels and Long-Term Blood Pressure Variability in Military Young Males: The CHIEF Study

**DOI:** 10.3390/ijerph17176094

**Published:** 2020-08-21

**Authors:** Pang-Yen Liu, Yu-Kai Lin, Kai-Wen Chen, Kun-Zhe Tsai, Yen-Po Lin, Eiki Takimoto, Gen-Min Lin

**Affiliations:** 1 Department of Cardiovascular Medicine, School of Medicine, University of Tokyo, Tokyo 113-0033, Japan; liupydr@gmail.com (P.-Y.L.); etakimo1@jhmi.edu (E.T.); 2 Department of Internal Medicine, Tri-Service General Hospital and National Defense Medical Center, Taipei 114, Taiwan; 3 Department of Medicine, Hualien Armed Forces General Hospital, Hualien 971, Taiwan; yukai0907@ndmctsgh.edu.tw (Y.-K.L.); az0127@gmail.com (K.-W.C.); stupidgrandpa@yahoo.com.tw (K.-Z.T.); 4 Department of Neurology, Tri-Service General Hospital, National Defense Medical Center, Taipei 114, Taiwan; 5 Department of Critical Care Medicine, Taipei Tzu-Chi General Hospital, New Taipei City 231, Taiwan; b101093018@tmu.edu.tw; 6 Department of Preventive Medicine, Northwestern University Feinberg School of Medicine, Chicago, IL 60611, USA

**Keywords:** alanine transaminase, aspartate transaminase, blood pressure variability, young males

## Abstract

*Background*: An inverse relationship of serum liver transaminases and mortality might be due to better blood pressure control in hypertensive patients. Whether it holds true regarding such an association for long-term blood pressure variability (BPV) in those without antihypertensive therapy is unclear. *Methods*: A population of 1112 military males without antihypertensive medications, aged 32 years, was collected from a retrospective longitudinal study in Taiwan. Serum liver aspartate and alanine transaminase (AST and ALT) levels were obtained from a 12 h-fast blood sample of each participant. BPV was assessed by standard deviation (SD) and average real variability (ARV) of systolic and diastolic blood pressure (SBP and DBP), respectively across 4 visits during the study period (2012–2014, 2014–2015, 2015–2016, and 2016–2018). Multivariable linear regression analysis was utilized to determine the association adjusting for demographics, anthropometric indexes, SBP, DBP, and lipid profiles. *Results*: In the unadjusted model, ALT was significantly and positively correlated with SD_DBP_ and ARV_DBP_ (β (standard errors) = 0.36 (0.16) and 0.24 (0.12), respectively), and so was AST (β = 0.19 (0.08) and 0.14 (0.06), respectively). All the associations were insignificant with adjustments. However, ALT was significantly and negatively correlated with SD_SBP_ and ARV_SBP_ (β = −0.35 (0.14) and −0.25 (0.11), respectively) and so was AST (β = −0.14 (0.07) and −0.12 (0.06), respectively) with adjustments. *Conclusion*: Our findings suggested that serum liver transaminases were negatively correlated with long-term systolic BPV in young male adults without antihypertensive therapy, and the clinical relevance needs further investigations.

## 1. Introduction

Several hepatobiliary biomarkers have been reported with an association with cardiovascular disease (CVD), disability, and all-cause deaths [1,2,3,4,5,6,7,8]. Numerous population-based reports [1,2,3,4] and a meta-analysis [5] consistently showed that people who had elevated serum concentrations of gamma-glutamyl transferase (GGT) or alkaline phosphate (ALP) had higher risk of CVD and all-cause deaths. Potential mechanisms underlying the relationship between elevated GGT and increased risk of CVD have been proposed. GGT has proinflammatory and pro-oxidant properties, possibly promoting the atherosclerotic process [9]. In addition, GGT is directly involved in atheromatous plaque formation as well [10]. With regard to ALP, it is proinflammatory, and can increase bone metabolism which contributes to vascular calcification, and impair vascular homoeostasis, leading to occurrence of CVD and related deaths [7,8].

In contrast, some hepatobiliary biomarkers have been revealed with beneficial effect or no influence on the cardiovascular system [3,11,12,13,14,15]. To our knowledge, bilirubin is a potent antioxidant and can suppress the oxidation of low-density lipoprotein cholesterol in humans [11,12]. In many population-based studies, serum bilirubin concentrations have been associated with lower risk of incident hypertension, coronary heart disease, and deaths [13,14,15]. However, regarding serum alanine transaminase (ALT) and aspartate transaminase (AST), the most two commonly used biomarkers for assessing hepatic injury in clinical practice, the associations with risk of CVD and deaths were inconsistently found higher, lower, or neutral in the general population [1,3,4,5,6].

Elevated serum liver transaminase concentrations are prevalent in people with viral hepatitis, toxin exposure such as alcohol intake, or nonalcoholic fatty liver disease [16,17,18]. Except chronic viral hepatitis [19], most of these risk factors are highly related to metabolic abnormalities, such as obesity, dyslipidemia, and prediabetes and prehypertension [20,21]. It remains unclear why some studies demonstrated that those with higher serum liver transaminases had lower risk of CVD or deaths, whereas others showed inverse results. Blood pressure variability (BPV), which has been associated with incidence of CVD, is classified according to the time-frame as short-term fluctuations of blood pressure within a 24-h period (beat-to-beat, minute-to-minute, hour-to-hour, and day-to-night changes, and long-term fluctuations over more-prolonged periods of time (days, weeks, months, and years) [22]. In a previous study [23], among hypertensive patients on antihypertensive treatment, higher serum ALT was associated with better long-term blood pressure control, explaining for the reduced CVD mortality risk. It is possible that serum liver transaminase may affect long-term variation of blood pressure in the general population. Therefore, we examined the association of serum AST and ALT with long-term blood BPV in a cohort of military young males.

## 2. Materials and Methods

### 2.1. Study Population

A cohort of 1112 healthy military males, averaged 32.2 years of age (18–40 years) and without taking any antihypertensive therapy, was retrospectively collected from the cardiorespiratory fitness and hospitalization events in armed forces (CHIEF) study in eastern Taiwan for the analysis [24]. All participants received a comprehensive health examination for requiring information of demographic data, past medical history of chronic viral hepatitis B or C, and tobacco smoking status (current vs. former and never), alcohol consumption status (current vs. former and never) and physical activity. A physical examination and blood biochemical tests were performed every two years from 2012 to 2018 (2012–2014, 2014–2015, 2015–2016, and 2016–2018) in the Hualien Armed Forces General Hospital.

### 2.2. Measurements of Anthropometric and Biochemical Blood Tests

Anthropometric measurements of body height and weight of each participant were performed in a standing position. Body mass index was defined as a ratio of body weight (kg) divided by body height squared (m^2^). Waist circumference was measured midway between the lower rib margin and iliac crest at standing position. Abdominal obesity was defined as waist circumference ≥90 cm for male adults. Blood biochemical tests for concentrations of triglycerides, total cholesterol, high-density lipoprotein, fasting glucose, and AST and ALT were enzymatically measured on an auto analyzer (AU640, Olympus, Kobe, Japan). All blood samples were collected by several experienced technicians at the same blood drawing station after an overnight 12-h fast for the participants [25,26,27,28,29,30].

### 2.3. Measurements of Long-Term BPV

**Figure 1:**
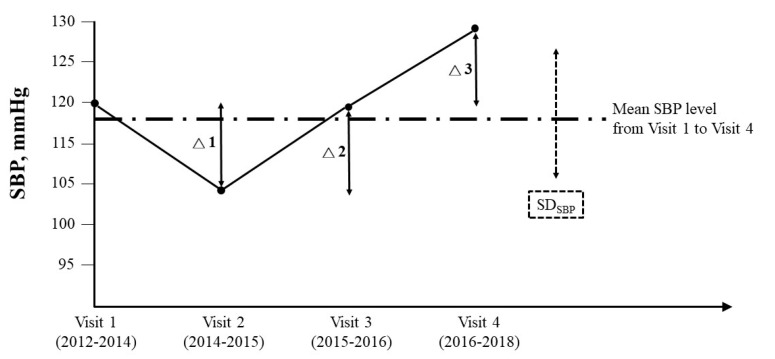
Long-term blood pressure variability (BPV) was assessed by standard deviation and average real variability across 4 visits in the study period (2012–2018). Standard deviation (SD)_SBP_ and average real variability (ARV)_SBP_ were respectively illustrated by dotted and solid lines. ARV_SBP_ is an average of absolute differences between successive systolic blood pressure measurements, and it takes the order of measurements into account (|Δ1| + |Δ2| + |Δ3|)/3).

Theophylline or caffeine-containing materials were forbidden for a 12-h fast of each participant before hospital visit. Measurements of systolic and diastolic blood pressure (SBP and DBP, respectively) of each participant was performed once over the right upper arm in a sitting position after a rest for at least 15 min, by a FT-201 automated blood pressure monitor (Parama-Tech Co Ltd., Fukuoka, Japan) at each visit [31,32]. Long-term BPV was assessed by standard deviation (SD_SBP_ and SD_DBP_) and average real variability (ARV_SBP_ and ARV_DBP_) across 4 visits in the study period (2012–2018). SD_SBP_ and ARV_SBP_ were respectively represented by dotted and solid lines in Figure 1. ARV is an average of the sum of absolute differences between successive blood pressure measurements, and it takes the order of measurements into account (|Δ1| + |Δ2| + |Δ3|)/3) [24,33,34].

### 2.4. Statistical Analysis

The characteristics of participants who had serum ALT ≥ 30 U/L, the upper limit of normal levels of serum ALT [35], (n = 254) and those who had serum ALT < 30 U/L (n = 858) were expressed as mean ± SD and compared by two-sample t-test for continuous data, and presented as numbers (%) and compared by chi-square test for categorical data. In addition, the characteristics of participants who had serum AST ≥ 30 U/L, the upper limit of normal levels of serum AST [35], (n = 118) and those who had serum AST < 30 U/L (n = 994) were compared in the same manner. In order to reveal the distribution of BPV for all study participants, the range of each BPV index was divided by quantile. Differences in serum liver transaminase levels among the quantile groups were assessed using analysis of covariance (ANCOVA) with adjustment for the baseline SBP and DBP levels. Unadjusted and multivariable adjusted linear regression were used to determine the relationship of serum AST and ALT levels with long-term BPV in the overall cohort and those without current alcohol intake (n = 681). In model 1, the baseline SBP and DBP levels (2012–2014) were initially adjusted. In model 2, age, body mass index, abdominal obesity, smoking status, alcohol intake status, serum triglycerides, fasting glucose, total cholesterol, high-density lipoprotein cholesterol, and physical activity (average weekly exercise frequency in past 3 months) were additionally adjusted. This study was approved by the Institutional Review Board of the Mennonite Christian Hospital (No. 16-05-008) in Hualien, Taiwan and written informed consent was obtained from all participants. A *p*-value < 0.05 was considered significant. SAS statistical software (SAS version 9.4; SAS Institute, Cary, NC, USA) was used for all statistical analyses.

## 3. Results

Table 1 reveals the baseline characteristics of the study participants (2012–2014). The males with higher serum ALT had similar heart rate, physical activity, and prevalence of current smokers and current alcohol consumers compared with those with lower serum ALT. However, the males with higher serum ALT had greater age, SBP, DBP, body mass index, waist circumference, fasting glucose, serum triglycerides, total cholesterol and serum AST, and lower high-density lipoprotein cholesterol. There were no participants reported with chronic viral hepatitis. Supplementary Table S1 shows similar patterns for the baseline profiles of the study participants classified by serum AST.

**Figure 2:**
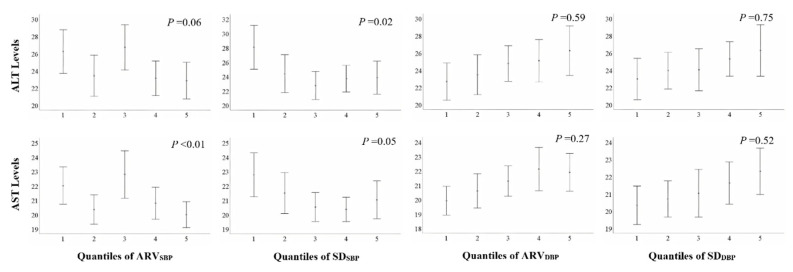
Serum liver transaminase levels in quantiles of average real variability (ARV)_BP_ and standard deviation (SD)_BP_. Bars represent means (95% confidence intervals) with adjustment for systolic blood pressure (SBP) and diastolic blood pressure (DBP). *p*-values were calculated by ANCOVA.

Figure 2 shows mean (95% confidence intervals) levels of serum AST and ALT with adjustment for the baseline SBP and DBP in quantiles of SD_BP_ and ARV_BP_. In general, higher SD_SBP_ and ARV_SBP_ were significantly associated with lower mean levels of serum AST and ALT, except a borderline significant association between ARV_SBP_ and mean levels of serum ALT (*p* = 0.06). In contrast, there were no significant relationships of diastolic BPV indexes with mean levels of serum AST and ALT with adjustment.

The results of multivariable linear regression analysis for the overall cohort are revealed in Table 2. In the unadjusted model, serum ALT and AST were positively and significantly correlated with ARV_DBP_ (*β* and standard errors (SE) = 0.24 (0.12) and 0.14 (0.06), respectively) and SD_DBP_ (*β* = 0.36 (0.16) and 0.19 (0.08), respectively). However, serum ALT and AST were negatively and borderline significantly correlated with ARV_SBP_ (*β* = −0.22 (0.12) and −0.11 (0.06), respectively) and SD_SBP_ (*β* = −0.28 (0.15) and −0.13 (0.08), respectively). With adjustment for the baseline SBP and DBP in model 1, the positive correlations of serum ALT and ALT with diastolic BPV indexes reduced and were insignificant, and so were in the fully adjusted model 2. On the contrary, the inverse correlations of serum ALT and AST with ARV_SBP_ in model 1 turned to be significant, and were consistently significant in model 2 (*β* = −0.25 (0.11) and −0.12 (0.06), respectively). Similarly, the inverse correlations of serum ALT and AST with SD_SBP_ were significant in model 1, and remained significant in model 2 (*β* = −0.35 (0.14) and −0.14 (0.07), respectively). In Supplementary Table S2, the results of multivariable linear regression analysis for those without current alcohol intake were in line with that for the overall cohort. In model 2, serum ALT was significantly and inversely correlated with ARV_SBP_ and SD_SBP_ (*β* = −0.36 (0.14) and −0.49 (0.19), respectively), and serum AST was borderline significantly and inversely correlated with ARV_SBP_ and SD_SBP_ (*β* = −0.13 (0.07) and −0.17 (0.09), respectively).

## 4. Discussion

Our main findings were that in military young males without antihypertensive therapy, those males with elevated levels of serum ALT (≥30 U/L) had higher baseline SBP and DBP compared with those with normal levels of serum ALT (<30 U/L), which were in line with the results in several previous reports [31,32,33,34,35]. Although there was a positive association of liver transaminase concentrations with long-term diastolic BPV, the association could be explained by the baseline SBP, DBP, and other potential covariates. In contrast, an inverse association of serum ALT and AST concentrations with long-term systolic BPV was present independently of the baseline blood pressure and all covariates adjustments, and was not altered in those without current alcohol intake.

To our knowledge, current evidence regarding a relationship between elevated serum ALT and higher blood pressure were observed in the general population regardless of young or old age [36,37,38,39]. However, the blood pressure association for serum AST was less than that for serum ALT, possibly due to contamination of AST in blood circulation from the sources other than liver such as muscle or gastrointestinal tracts [36,37]. In addition, consumption of alcoholic beverages has been reported as a possible confounder. Fu et al. found that alcohol intake might reduce the positive association between higher serum ALT levels within normal ranges and increased arterial stiffness and elevated blood pressure [40]. However, some studies showed that alcohol intake would not alter the positive association between serum liver transaminase levels and blood pressure in the middle- and old-aged individuals [37,41]. Whether total accumulated amount of alcohol intake may mediate the relationship has not be confirmed and needs further investigations.

With regard to the studies reporting a positive association between serum ALT and risk of mortality, the results were obtained from the studies for middle or old-aged individuals who had multiple cardiovascular risk factors and without excluding chronic viral hepatitis [2,4,6]. In addition, the positive relationship was mainly observed in the subgroups of those with Asian ethnicity, male sex, or those with low body mass index [2,4,6]. However, several studies including a meta-analysis paradoxically revealed an inverse or null association of serum ALT levels with incident CVD and related mortality in the general population [3,5,6]. Possible mechanisms for the inverse relationship with mortality have been proposed. First, low ALT levels might reflect a poor nutritional status and low body mass [3]. In addition, low ALT might be associated with reduced liver size and blood flow in the elderly who had lower productions of protein and greater susceptibility to toxins, possibly increasing the risk of mortality [42,43].

However, it remains unclear regarding the inverse relationship between serum ALT and risk of CVD [6,22]. Though McCallum et al. have found that the inverse result might be due to higher serum ALT with better long-term blood pressure control in hypertensive patients on medical treatment [22], the biological mechanisms for the inverse association between serum ALT levels and long-term blood pressure changes are still vague [22]. The longitudinal SBP changes related to alteration in pharmaco-kinetic or pharmacodynamic parameters are likely reflected in serum ALT levels [22]. In their study, the effect of serum ALT on longitudinal SBP changes were prominent in subgroups of older ages or overweight or consumed alcohol [22]. Instead of being with multiple comorbidities, our report was the first study using healthy young male adults who did not use antihypertensive medications. It clearly clarified that the positive association between serum ALT levels and long-term diastolic BPV, a CVD risk factor, could be accounted for the baseline blood pressure levels. As was known, blood pressure levels were the major determinant of BPV and have been proposed to explain the association between BPV and CVD in some studies [44,45]. By contrast, the inverse association with long-term systolic BPV was independent of all the adjusted confounders, which was in line with the findings by McCallum et al. Although light or moderate alcohol intake may increase serum liver enzymes and reduce risk of CVD death and short-term BPV, possibly by lowering aortic stiffness [46,47,48], the inverse relationship of serum ALT and AST with long-term systolic BPV was also observed in participants without alcohol intake and the dose effect of alcohol consumption in active drinkers could not be assessed for lack of relevant information in this study.

There were several strengths in this study. First, the physical and laboratory examinations were all performed in a strict manner, and the whole procedures were standardly performed in the same referral military hospital. Second, since the daily lifestyle of military such as the diet intake and physical training were unified, many unmeasured factors had been controlled at baseline. By contrast, there were some limitations in this study. First, the results were merely obtained from male subjects and might not be applied to female subjects. Second, although many covariates at baseline were adjusted in the model, we could not completely avoid the possibility of existing potential confounders to result in a bias, such as information for the amount of weekly alcohol intake which was not available from the questionnaire. Third, although we excluded participants with chronic viral hepatitis B or C by a self-report, we could not guarantee the data clean despite the prevalence of chronic viral hepatitis B less than 3% in the military in Taiwan [16].

In conclusion, our findings suggested that serum ALT and AST concentrations were significantly and inversely correlated with long-term systolic BPV among healthy military young males without taking any antihypertensive medications and free of history of chronic viral hepatitis B or C, as well as in those without active alcohol intake. These novel findings might provide support for the inverse relationship of serum liver transaminases with risk of CVD and mortality found in previous studies. However, it remains unclear regarding the mechanisms of the inverse association for serum ALT and AST with long-term systolic BPV, and we require further study to determine which unmeasured bias may account for the reverse epidemiology.

## Figures and Tables

**Table 1:** Clinical characteristics of the study cohort.

Variables	ALT ≥ 30 U/L (N = 254)	ALT < 30 U/L (N = 858)	*p*-Value
Age (yr)	32.71 ± 3.74	32.07 ± 3.91	0.02
BMI (kg/m^2^)	26.81 ± 2.48	24.64 ± 2.83	<0.001
Waist circumference (cm)	88.51 ± 6.12	83.10 ± 7.09	<0.001
SBP (mmHg)	121.31 ± 13.40	117.47 ± 13.26	<0.001
DBP (mmHg)	73.22 ± 10.60	71.12 ± 9.89	0.004
Heart rate (beats/minute)	75.74 ± 11.16	74.81 ± 10.43	0.22
Serum triglycerides (mg/dL)	164.01 ± 136.37	110.88 ± 91.09	<0.001
FPG (mg/dL)	96.63 ± 16.20	93.87 ± 12.43	0.004
Total cholesterol (mg/dL)	191.93 ± 37.36	176.67 ± 30.43	<0.001
HDL-C (mg/dL)	45.82 ± 9.69	48.66 ± 9.92	<0.001
ALT (U/L)	48.04 ± 24.40	17.55 ± 5.74	<0.001
(Minimum–Maximum)	(30–213)	(5–29)	
AST (U/L)	30.50 ± 13.54	18.45 ± 5.03	<0.001
(Minimum–Maximum)	(16–124)	(10–57)	
Physical activity			
Never or occasionally	42 [16.5]	143 [16.7]	0.99
1–2 times/week	98 [38.6]	329 [38.3]	
≥3–5 times/week	114 [44.9]	386 [45.0]	
Current alcohol drinker (n [%])	121 [47.6]	396 [46.2]	0.67
Current smoker (n [%])	99 [39.0]	332 [38.7]	0.93

Continuous variables are expressed as mean ± standard deviation and categorical variables as number (percentage). Abbreviations: ALT, alanine aminotransferase; AST, aspartate aminotransferase; BMI, body mass index; FPG, fasting plasma glucose; HDL-C, high density lipoprotein cholesterol.

**Table 2:** Association of serum alanine transaminase (ALT) and aspartate transaminase (AST) levels with long-term blood pressure variability in multivariable liner regression.

BPV Indexes	Unadjusted			Model 1			Model 2		
	*β* (SE)	*p*-Value	R^2^, %	*β* (SE)	*p*-Value	R^2^, %	*β* (SE)	*p*-Value	R^2^, %
ALT levels									
ARV_SBP_	−0.217 (0.115)	0.05	0.3	−0.225 (0.114)	0.04	1.8	−0.253 (0.108)	0.01	13.7
ARV_DBP_	0.240 (0.122)	0.04	0.4	0.157 (0.123)	0.20	1.6	0.087 (0.116)	0.45	13.3
SD_SBP_	−0.277 (0.149)	0.06	0.3	−0.297 (0.148)	0.04	1.8	−0.345 (0.140)	0.01	13.8
SD_DBP_	0.362 (0.161)	0.02	0.5	0.236 (0.164)	0.15	1.7	0.151 (0.155)	0.33	13.4
AST levels									
ARV_SBP_	−0.114 (0.059)	0.05	0.3	−0.117 (0.059)	0.04	1.9	−0.115 (0.057)	0.04	9.2
ARV_DBP_	0.139 (0.063)	0.02	0.4	0.097 (0.064)	0.12	1.8	0.071 (0.062)	0.25	9.0
SD_SBP_	−0.127 (0.077)	0.09	0.2	−0.135 (0.076)	0.07	1.9	−0.144 (0.074)	0.05	9.2
SD_DBP_	0.187 (0.083)	0.02	0.5	0.123 (0.085)	0.14	1.8	0.096 (0.082)	0.24	9.0

Data are presented as *β* (SE, standard errors) using Pearson’s correlation coefficient for the association of serum ALT and AST levels with long-term blood pressure variability (BPV) with adjustments in Model 1: systolic blood pressure (SBP) and diastolic blood pressure (DBP) adjustments; Model 2: the covariates in Model 1, age, body mass index, waist circumference ≥90 cm, serum triglycerides, total cholesterol, high-density lipoprotein, physical activity, current alcohol consumer, and current smoker.

## Supplementary Materials

The following are available online at https://www.mdpi.com/1660-4601/17/17/6094/s1, Table S1: Clinical Characteristics of Study Cohort, Table S2: Association of Serum ALT and AST with Long-Term Blood Pressure Variability in Participants Without Current Alcohol Intake in Multivariable Liner Regression.
