# Supplementary material for: Association of Liver Transaminase Levels and Long-Term Blood Pressure Variability in Military Young Males: The CHIEF Study
Source: Int J Environ Res Public Health. 2020 Aug 21;17(17):6094. doi: 10.3390/ijerph17176094 (PMC7504620; doi:10.3390/ijerph17176094)
Supplement: Supplementary file 1 [file ijerph-17-06094-s001.pdf]

Table S1. Clinical Characteristics of Study Cohort.

	AST ≥40 U/L (N = 44)	AST <40 U/L (N = 1068)	*p*-Value
Age (yr)	32.59 ± 3.34	32.20 ± 3.91	0.51
BMI (kg/m^2)	26.89 ± 2.60	25.07 ± 2.89	<0.01
SBP (mmHg)	121.84 ± 13.26	118.21 ± 13.38	0.07
DBP (mmHg)	74.73 ± 10.17	71.48 ± 10.08	0.03
Heart rate (beats/minute)	73.84 ± 12.21	75.08 ± 10.54	0.45
FPG (mg/dL)	94.66 ± 12.82	94.49 ± 13.46	0.93
Total cholesterol (mg/dL)	198.55 ± 42.40	179.40 ± 32.11	<0.01
HDL-C (mg/dL)	46.86 ± 9.88	48.06 ± 9.95	0.43
ALT (U/L) (Minimum–Maximum)	77.30 ± 41.43 (18–213)	22.34 ± 12.23 (5–92)	<0.01
AST (U/L) (Minimum–Maximum)	54.64 ± 18.14 (40–124)	19.82 ± 5.41 (10–39)	<0.01
Physical activity			
Never or occasionally	8 [18.2]	177 [16.6]	0.95
1–2 times/week	17 [38.6]	410 [38.4]	
≥3 times/week	19 [43.2]	481 [45.0]	
Current smoker, %	16 [36.4]	415 [38.9]	0.73
Current alcohol intake, %	22 [50.0]	495 [46.3]	0.63

Continuous variables are expressed as mean ± standard deviation and categorical variables as number (percentage). Abbreviations: ALT, alanine aminotransferase; AST, aspartate aminotransferase; BMI, body mass index; FPG, fasting plasma glucose; HDL-C, high density lipoprotein cholesterol.

Table S2. Association of Serum ALT and AST with Long-Term Blood Pressure Variability in Participants Without Current Alcohol Intake in Multivariable Liner Regression.

	Unadjusted			Model 1			Model 2		
	β (SE)	*p*-Value	R^2, %	*β* (SE)	*p*-Value	R^2, %	*β* (SE)	*p*-Value	R^2, %
ALT levels									
ARV_{SBP}	−0.299 (0.152)	0.04	0.7	−0.318 (0.151)	0.03	2.1	−0.354 (0.144)	0.01	13.6
ARV_{DBP}	0.043 (0.168)	0.80	0.0	−0.035 (0.170)	0.83	1.4	−0.063 (0.161)	0.69	12.8
SD_{SBP}	−0.376 (0.200)	0.06	0.6	−0.394 (0.199)	0.04	2.0	−0.477 (0.189)	0.01	13.7
SD_{DBP}	−0.002 (0.219)	0.99	0.0	−0.130 (0.223)	0.55	1.4	−0.174 (0.211)	0.41	12.9
AST levels									
ARV_{SBP}	−0.113 (0.073)	0.12	0.4	−0.123 (0.073)	0.09	1.8	−0.128 (0.071)	0.07	9.2
ARV_{DBP}	0.071 (0.081)	0.37	0.1	0.035 (0.082)	0.66	1.3	0.018 (0.079)	0.82	8.7
SD_{SBP}	−0.133 (0.096)	0.16	0.3	−0.142 (0.096)	0.13	1.7	−0.167 (0.093)	0.07	9.2
SD_{DBP}	0.040 (0.106)	0.70	0.0	−0.020 (0.107)	0.85	1.3	−0.042 (0.104)	0.69	8.7

Data are presented as *β* (SE, standard errors) using Pearson's correlation coefficient for Model 1: systolic blood pressure (SBP) and diastolic blood pressure (DBP) adjustments; Model 2: the covariates in Model 1, age, body mass index, total cholesterol, high-density lipoprotein, physical activity, current alcohol consumer and current smoker adjustments.
